# Development and Characterization of Biogenic Hydroxyapatite Coatings Derived from Crab Shell Waste on Ti6Al4V Substrates

**DOI:** 10.3390/ma18225222

**Published:** 2025-11-18

**Authors:** Yago Antonio de Lima Guedes, Maurício Maia Ribeiro, Douglas Santos Silva, Raí Felipe Pereira Junio, Roberto Paulo Barbosa Ramos, Sergio Neves Monteiro, Elza Monteiro Leão Filha, Jean da Silva Rodrigues

**Affiliations:** 1 Materials Engineering Program, Federal Institute of Education, Science and Technology of Pará—IFPA, Avenida Almirante Barroso, 1155, Marco, Belém CEP 66093-020, PA, Brazil; yagoguedes54@gmail.com (Y.A.d.L.G.); roberto.ramos@ifpa.edu.br (R.P.B.R.); elza.filha@ifpa.edu.br (E.M.L.F.); jean.rodrigues@ifpa.edu.br (J.d.S.R.); 2 Federal Institute of Education, Science and Technology of Pará—IFPA, Estrada do Icuí Guajará, Ananindeua CEP 67125-000, PA, Brazil; mauricio.maia@ifpa.edu.br; 3 Military Institute of Engineering—IME, Department of Materials Science, Praça General Tibúrcio, 80, Praia Vermelha, Urca, Rio de Janeiro CEP 22290-270, RJ, Brazil; raivsjfelipe@ime.eb.br (R.F.P.J.); sergio.neves@ime.eb.br (S.N.M.)

**Keywords:** biogenic hydroxyapatite, crab shell waste, sustainable biomaterials, titanium alloy Ti6Al4V

## Abstract

In this work, we developed and characterized a hydroxyapatite (HA) ceramic coating derived from *Ucides cordatus* crab-shell waste and applied it onto Ti–Al–V titanium substrates for biomedical use. Substrate analysis confirmed an α + β two-phase microstructure and Rockwell C hardness of ~35 HRC; optical emission spectrometry indicated a non-conforming Ti–6Al–4V composition (Al slightly above and V slightly below ASTM F136-18 limits), with expected α-phase predominance. Aqueous synthesis of biogenic HA used CaO (from 800 °C calcined shells) reacted with β-tricalcium phosphate (β-Ca_3_(PO_4_)_2_), followed by deposition onto Ti–Al–V surfaces prepared with or without a thermochemical treatment that homogenized roughness (Ra ≈ 0.587 µm). The coatings were continuous, ~95–98 µm thick, and showed good qualitative adhesion. Scanning Electron Microscopy (SEM) revealed porous, nanocrystalline, acicular morphologies typical of biogenic apatite’s. Energy-Dispersive X-ray Spectroscopy (EDS) yielded Ca/P ≈ 1.85–1.88, while X-ray Fluorescence (XRF) indicated Ca-enrichment relative to stoichiometric HA. X-ray Diffraction (XRD) confirmed a predominantly hexagonal HA phase with high crystallinity. These results demonstrate a technically and environmentally feasible route to bioactive coatings using marine biowaste, aligning low-cost, local waste valorization with functional performance on titanium implants.

## 1. Introduction

Titanium and its alloys, particularly Ti6Al4V, are widely recognized in the biomedical field for their excellent combination of mechanical strength, corrosion resistance, and biocompatibility [1,2]. However, their intrinsically bioinert nature limits direct osseointegration, often resulting in insufficient bonding at the bone–implant interface. To overcome this limitation, the surface functionalization of titanium through the deposition of bioactive ceramic coatings—especially hydroxyapatite (HA, Ca_10_(PO_4_)_6_(OH)_2_)—has emerged as an effective strategy to enhance biological affinity and improve the long-term performance of metallic implants [3,4]. Recent studies have further demonstrated that HA-based coatings on titanium substrates significantly improve osteogenic responses, interfacial bonding, and early-stage osseointegration in orthopedic and dental implant applications, reinforcing their relevance in next-generation biomedical devices [5]. Advanced coating technologies such as hydrothermal treatment, plasma spraying, and electrophoretic deposition have enabled the fabrication of HA layers with improved adhesion, crystallinity, and tribological behavior on Ti6Al4V substrates [6,7,8,9].

Recent advances have also explored alternative strategies to enhance the performance of HA coatings, such as doping with bioactive ions (e.g., Sr^2+^, Zn^2+^, Mg^2+^), the incorporation of polymeric or metallic phases, and the use of hybrid deposition techniques. These approaches have been shown to improve crystallinity, adhesion strength, corrosion resistance, and osteogenic activity of HA-based coatings [5,6,7,8]. Furthermore, recent reviews have emphasized the transition toward sustainable and eco-efficient synthesis routes, highlighting the valorization of natural and marine resources, such as mollusk and crab shells, as calcium sources for the production of calcium phosphates and bioactive ceramics [10]. This growing body of research supports the global trend toward environmentally conscious biomaterial development and provides a strong scientific foundation for the present study. HA exhibits chemical composition and crystal structure similar to the mineral phase of bone, providing osteoconductivity, biocompatibility, and chemical stability under physiological conditions. Therefore, the development of HA coatings on titanium substrates represents one of the most promising approaches for improving bone integration and durability in orthopedic and dental implants [3,4].

Traditionally, HA used in biomedical applications is synthesized from high-purity chemical reagents such as synthetic calcium and phosphate salts. Although these routes yield well-defined crystalline phases, they rely on non-renewable resources, entail high production costs, and generate environmental impacts associated with reagent purification and waste disposal [10]. In contrast, the valorization of biological residues as raw materials for ceramic synthesis represents a sustainable alternative aligned with the principles of the circular economy. Marine exoskeletons—such as shells, corals, and crustaceans—are rich in calcium carbonate (CaCO_3_), which can be converted into calcium oxide (CaO) by calcination and subsequently transformed into HA through hydrothermal or wet-chemical processes [10,11,12,13]. This biogenic route not only mitigates the environmental burden associated with marine waste disposal but also produces materials naturally enriched with trace elements (e.g., Sr^2+^, Mg^2+^, Na^+^), which can enhance the biological activity of HA [10,11,12,13,14].

These ionic substitutions play specific roles in improving the biological performance of calcium HA. Strontium (Sr^2+^) can replace Ca^2+^ sites in the apatite lattice, promoting osteoblast proliferation and inhibiting osteoclast-mediated bone resorption, thus enhancing osteogenesis and reducing bone turnover. Magnesium (Mg^2+^), which also substitutes for Ca^2+^, increases lattice disorder and surface reactivity, accelerating apatite dissolution and favoring early bone mineralization. Sodium (Na^+^) incorporation contributes to charge compensation and the formation of A- and B-type carbonated apatite’s, which more closely resemble biological apatite and improve bioresorption and cell adhesion. Consequently, these minor ionic substitutions increase the overall biocompatibility and bioactivity of HA coatings, facilitating stronger and faster bone–implant integration [15,16].

In addition to its widespread use in coatings for metallic implants, HA has recently gained attention in emerging fields of biomedical engineering. It is currently employed in the fabrication of porous and bioactive structures for use in biological reactors, where its high surface area and chemical stability support cell proliferation and biomolecule immobilization. Moreover, HA powders and composites have been incorporated as fillers in additive manufacturing processes, including selective laser sintering (SLS) and fused deposition modeling (FDM), to produce customized scaffolds and bone substitutes with controlled porosity and mechanical properties. These new applications illustrate the growing versatility of HA in advanced bio fabrication technologies, reinforcing its relevance as a multifunctional biomaterial.

Beyond HA synthesis, the marine economy offers a vast range of materials and bioresources that can be converted into high-value products for human development. Recent studies have highlighted the potential of marine-derived minerals, biopolymers, and biological residues as renewable sources for functional materials [17,18]. Marine biowastes such as shells, corals, crustaceans, fish bones, and algae can be transformed into calcium phosphate ceramics, biocompatible polymers (e.g., chitosan and collagen), and nanostructured composites with applications in biomedicine, environmental remediation, catalysis, and energy storage. This circular approach exemplifies the principles of the blue economy, promoting the sustainable use of ocean resources to produce materials that enhance human health and technological advancement. Within this context, the utilization of crustacean exoskeletons to produce HA aligns with the broader goal of integrating marine resource valorization into sustainable development strategies.

Among marine-derived biomaterials, the exoskeleton of the uçá crab (*Ucides cordatus*), abundantly found along the Brazilian coastline, represents an underexplored calcium source with significant technological potential. Its use for HA synthesis offers an environmentally responsible and economically feasible alternative while fostering the development of sustainable, locally sourced materials. Similar strategies using crab or mollusk shells have successfully produced biogenic HA with hierarchical nanostructures and doped compositions beneficial for bone tissue regeneration [12,13,14,15,16,17,18,19]. Moreover, biogenic HA typically exhibits a nanocrystalline and porous morphology, increasing the specific surface area and favoring protein adsorption, secondary apatite nucleation, and cell adhesion—key characteristics for improved biological integration of biomedical implants [11,14,15,16,17,18,19,20].

Several techniques have been applied for HA deposition on metallic substrates, including plasma spraying, sol–gel processing, electrophoretic deposition, and hydrothermal methods [3,6,9,21]. Among these, the hydrothermal approach stands out due to its simplicity, low processing temperature, and ability to produce adherent and crystalline coatings without the need for high-energy equipment [6]. Additionally, thermochemical surface treatments applied to titanium substrates can increase surface energy and roughness, improving wettability and HA nucleation, which enhances coating adhesion and uniformity [4,7,9].

In this context, the present work proposes a sustainable route for obtaining biogenic HA (HACRAB) synthesized from calcined *Ucides cordatus* crab shell waste, followed by its deposition on Ti6Al4V metallic substrates via a hydrothermally assisted coating process. The study focuses on the relationship between surface treatment and the adhesion, morphology, and structural integrity of the resulting coating. Physicochemical, morphological, and structural characterizations—including X-ray fluorescence (XRF), scanning electron microscopy coupled with energy-dispersive X-ray spectroscopy (SEM/EDS), X-ray diffraction (XRD), and surface roughness analyses—were performed to evaluate the quality and crystallinity of the deposited layers.

The novelty of this research lies in the development of a biogenic HA coating entirely derived from marine waste, successfully applied to a titanium alloy substrate, demonstrating that sustainable materials can achieve structural and functional performance comparable to conventional synthetic coatings. This approach combines environmental responsibility, economic feasibility, and biomedical applicability, contributing to scientific and technological advances in the field of eco-efficient biomaterials and establishing a promising alternative for the production of bioactive coatings for next-generation metallic implants.

This research stands out for its innovative approach combining sustainable raw materials with biomedical functionality. Unlike conventional HA coatings synthesized from chemical precursors, this study demonstrates, for the first time, the successful use of *Ucides cordatus* crab shell waste as a calcium source for obtaining high-purity HA directly deposited on Ti6Al4V substrates. The originality of the work lies in integrating waste valorization, eco-efficient synthesis, and coating deposition into a single low-temperature process, providing an environmentally friendly and technically feasible route for bioactive surface functionalization of titanium implants.

Therefore, the objective of this study was to develop and characterize a ceramic coating of biogenic HA obtained from *Ucides cordatus* crab shell waste and applied to Ti6Al4V alloy substrates. The research aimed to evaluate the influence of thermochemical surface treatment on the morphology, chemical composition, crystallinity, and adhesion of the coating, thereby demonstrating the technical and environmental feasibility of the proposed route for the production of sustainable biomaterials for medical and dental applications.

## 2. Materials and Methods

### 2.1. Characterization of the Metallic Material

The characterization of the metallic substrates was carried out to confirm the composition, microstructural integrity, and mechanical properties of the material used as the base for the deposition processes. The Ti–6Al–4V alloy plates used as substrates were supplied by Metalaf Indústria e Comércio de Metais Ltd.a. (São Paulo, Brazil), a certified distributor of titanium alloys for mechanical and biomedical applications. The material was received in the as-supplied condition, corresponding to a mill-rolled sheet with nominal dimensions of 20 mm × 20 mm × 3 mm, and was used without additional heat treatment prior to surface preparation. For this purpose, the study encompassed the analysis of three fundamental aspects: chemical composition, microstructure, and hardness. The chemical analysis aimed to identify and quantify the alloying elements, ensuring conformity with the nominal composition of the Ti6Al4V alloy. The microstructural characterization was performed using optical and electron microscopy techniques, enabling the observation of phase distribution, grain morphology, and possible heterogeneities within the material. Finally, the hardness evaluation was employed as a complementary parameter to verify the uniformity of the substrate and to correlate mechanical behavior with the observed microstructure. The results obtained were subsequently compared with data reported in the specialized literature, in order to validate the identification of the metallic alloy and ensure that the substrate employed exhibits properties consistent with those of the reference material adopted in this study.

#### 2.1.1. Chemical Analysis

The chemical analysis was performed on two specimens of the titanium alloy used in this study, with the purpose of confirming its elemental composition and verifying conformity with the standard specifications for the Ti6Al4V alloy. The test was conducted using an optical emission spectrometer (OES), model Foundry Master Xpert, manufactured by Oxford Instruments, available at the Laboratory of Metallic Materials Characterization (LCAM), Faculty of Mechanical Engineering, Institute of Technology (ITEC), Federal University of Pará (UFPA). To ensure accuracy and reproducibility of the measurements, the spectrometer was purged with compressed argon gas at a pressure of 3.0 bar for a period of 4 h, using a vacuum pump to eliminate residual impurities within the analysis chamber. Data acquisition and processing were carried out on a computer equipped with the Waslab analytical software, configured with the Ti_100 analytical program, which is specifically designed for the analysis of titanium alloys. For each prepared surface of the two samples, four sequential burns were performed to obtain representative elemental composition data. The results were subsequently evaluated through analysis of variance (ANOVA), considering a confidence level of 99.7%, in order to assess the statistical consistency of the measurements and confirm the chemical homogeneity of the analyzed material.

#### 2.1.2. Metallographic Tests

The metallographic tests were conducted in accordance with the procedures established by the ASTM E3-11 [22], which provides guidelines for the preparation, polishing, and chemical etching of metallic samples for microstructural examination. The process began with the cutting of the samples using a metallographic cutter equipped with an abrasive disc suitable for hard metals, in order to minimize overheating and the introduction of residual stresses. Due to the high hardness of titanium, slight dimensional irregularities were observed after cutting. Considering the small size of the metallic substrates, it was necessary to mount the specimens in phenolic powder using an electro-hydraulic mounting press, to facilitate handling during the subsequent grinding stages. The grinding process was carried out on a manual grinding machine, using abrasive papers of progressively finer grit sizes (80, 120, 320, 400, 600, and 1200 mesh) to achieve gradual surface leveling. This step was followed by mechanical polishing on a motorized metallographic polisher, employing a polishing cloth and a 0.5 μm alumina suspension until a mirror-like surface finish was obtained. The polished surfaces were then chemically etched by immersion in Kroll’s reagent, composed of 1–3 mL HF and 2–6 mL HNO_3_ in 1000 mL of H_2_O, according to the recommendations of ASM International (1985) [23]. After etching, the samples were rinsed with 70% ethyl alcohol and dried with warm air to prevent surface oxidation. The final cleaning was performed using an ultrasonic cleaner (model CD-4820), with a solution of absolute ethyl alcohol (99.99%) and analytical-grade acetone (P.A.), ensuring the complete removal of residues and contaminants. The microstructural observations were carried out using an AXIO Lab.A1 optical microscope (Carl Zeiss), located at the Inspection Laboratory of the Mechanical Engineering Technical Program, Federal Institute of Pará (IFPA)—Belém Campus.

#### 2.1.3. Scanning Electron Microscopy (SEM)

Scanning Electron Microscopy (SEM) was employed to complement the optical analyses, providing high-resolution images and a more detailed visualization of the microstructural features, thereby enabling a more accurate assessment of the material’s morphological characteristics and metallurgical transformations. In addition to morphological observation, the equipment was also used to perform Energy-Dispersive X-ray Spectroscopy (EDS), a technique that allows the identification and semi-quantitative analysis of chemical elements, based on the characteristic X-ray energies emitted by atoms excited under the electron beam. The analyses were carried out using a Vega 3 Scanning Electron Microscope (TESCAN), located at the Metallography Laboratory of the Materials Engineering Program, Federal Institute of Pará (IFPA)—Belém Campus. Secondary electron images were acquired under the following operating conditions: beam current of 90 μA, constant accelerating voltage of 20 kV, and working distance of 15 mm.

Prior to SEM imaging, all metallographic samples were sputter-coated with a thin conductive gold–palladium (Au–Pd) layer using a Quorum SC7620 sputter coater. The deposited coating had an approximate thickness of 8–12 nm, which was sufficient to prevent charging artifacts and improve image resolution during high-magnification analysis.

Although no external calibration standards were used during the EDS session, it is important to clarify that the detector was fully calibrated according to the manufacturer’s factory protocol and subjected to routine internal energy calibration prior to analysis. The system automatically performs peak alignment checks using built-in reference lines (typically Cu Kα at 8.04 keV), ensuring that elemental identification remains accurate within the expected limits of a silicon-drift detector. Because no certified external standards were applied, the reported compositions should be interpreted as semi-quantitative values, with typical uncertainties on the order of ±1–3 wt.% for major elements. These accuracy levels are fully adequate for confirming the Ca-rich composition trends discussed in this work and for supporting comparative interpretations, although they are not intended as high-precision quantitative measurements.

#### 2.1.4. Hardness Test

The hardness test was performed on two specimens at the Inspection Laboratory of the Mechanical Engineering Technical Program, Federal Institute of Pará (IFPA)—Belém Campus, with the purpose of determining the material’s resistance to indentation and complementing the mechanical characterization of the metallic substrate. Measurements were carried out using a Rockwell electronic bench hardness tester, model TH300, manufactured by TIME Group Inc., equipped with a diamond cone (Brale) indenter. The test was conducted under an applied load of 150 kgf, in accordance with the procedures specified by the ASTM E18-20 [24], ensuring the reproducibility and metrological traceability of the results obtained.

### 2.2. Preparation of Organic Residues

To obtain the HA used for coating the metallic substrates, crab shells derived from post-consumer domestic waste were utilized, taking advantage of their high calcium carbonate (CaCO_3_) content. The preparation procedure followed the methodology described by [25], with adaptations made according to the available laboratory infrastructure. Initially, the residues were washed under running water and manually brushed to remove surface impurities and any adhering organic matter. After cleaning, the samples were air-dried under natural ventilation and lighting for 24 h, ensuring the complete removal of surface moisture. Although the cleaning procedure employed was essentially mechanical, consisting of washing, brushing, and natural drying, it was designed to remove superficial contaminants, organic residues, and adhering biological matter. While this approach ensures adequate cleanliness for laboratory-scale synthesis and characterization, it does not achieve the high chemical purity typically required for biomedical-grade reagents. Consequently, the HA obtained in this study should be regarded as a proof-of-concept material for sustainable synthesis, not yet intended for direct clinical application. Achieving medical-grade purity would require additional chemical purification steps, such as acid–base washing, dialysis, or multi-stage calcination under controlled atmospheres, as commonly reported for biomaterial precursors used in implantable systems. The grinding process was performed manually using a rubber hammer, applying controlled impacts to the shells wrapped in a cotton cloth to prevent material loss. This operation was repeated until the crushed particles reached dimensions suitable for placement in a refractory crucible used in the subsequent step. To convert the calcium carbonate (CaCO_3_) from the crab exoskeletons into calcium oxide (CaO), a calcination process was carried out according to the following reaction:

$$Ca{CO}_{3}\to CaO+{CO}_{2}$$

**Figure 1:**
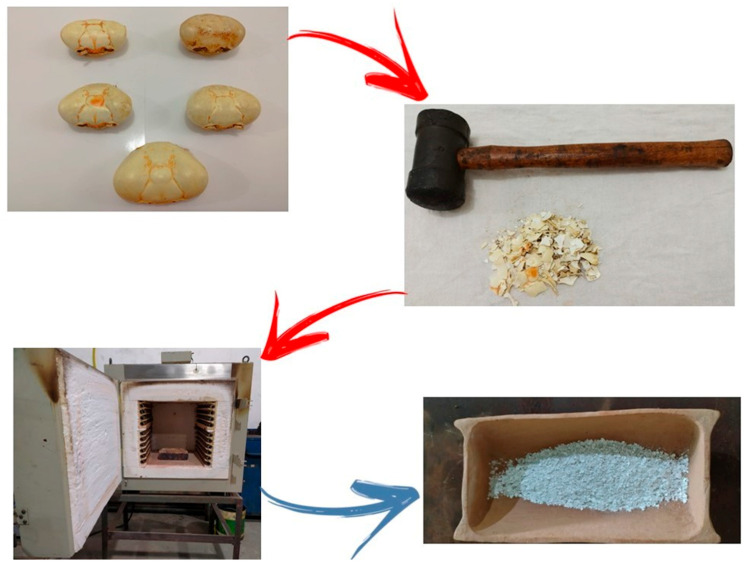
Stages of the preparation process of organic residues used for obtaining crab shell–derived HA (HACRAB): selection and cleaning of crab shells; manual crushing using a rubber hammer; calcination of the residues in a muffle furnace at 800 °C for 3 h; and calcined material with a powdery, grayish appearance, characteristic of calcium oxide (CaO) formation.

The process was performed in a muffle furnace, model 9612 by Jung, located at the Machining Laboratory of the Mechanical Engineering Technical Program, Federal Institute of Pará (IFPA)—Belém Campus. The calcination was conducted at 800 °C for 3 h, resulting in a grayish powder with an ash-like appearance, characteristic of calcium oxide formation. The phase composition of the calcined material was inferred from its color, texture, and reactivity rather than confirmed by direct X-ray diffraction (XRD) analysis. Although a diffraction pattern of the calcined *Ucides cordatus* crab shell was not obtained in this work, the formation of calcium oxide (CaO) as the predominant phase can be reasonably inferred based on the literature for similar marine biowaste precursors calcined at 800 °C. Previous studies have shown that crustacean shells subjected to thermal treatment above 750 °C exhibit complete decomposition of CaCO_3_ into CaO, with no residual calcite or aragonite peaks detected in XRD spectra [10,11,12,13]. The observed grayish coloration and the vigorous reactivity of the powder in water further support the conversion of CaCO_3_ to CaO, which served as the calcium source for HA synthesis. Figure 1 shows the process of preparing organic waste.

### 2.3. Deposition of the HA Layer on the Surface of Metallic Samples

**Figure 2:**
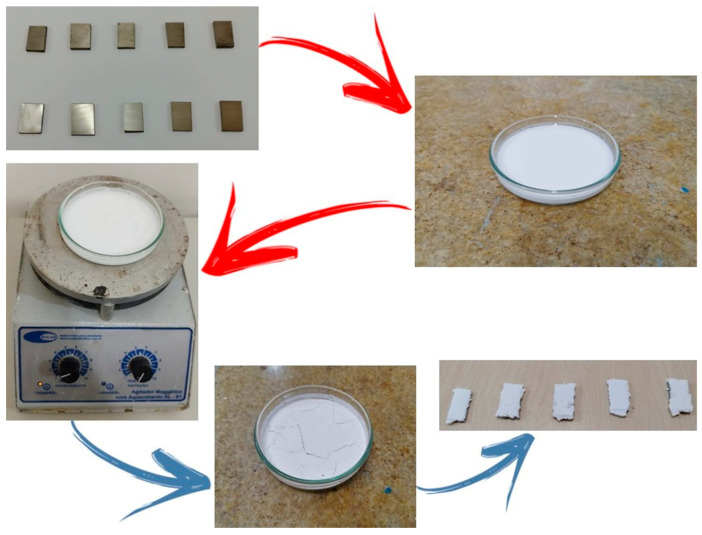
Stages of the deposition process of crab shell–derived HA (HACRAB) coating on titanium metallic substrates: preparation and selection of the metallic samples; HA suspension prepared for the deposition process; magnetic stirring with heating for suspension homogenization; formation and drying of the ceramic layer; and titanium samples coated with the HACRAB layer after the deposition process.

The deposition of the ceramic HA layer was carried out on a total of ten metallic samples. Among these, five samples were previously subjected to mechanical grinding followed by a thermochemical treatment to promote surface activation and enhance HA nucleation, while the remaining five samples did not undergo any surface modification or treatment and were used as the reference group. The preparation and deposition procedures were performed, with appropriate adaptations, according to the methodology described by [22], ensuring process reproducibility and comparability between treated and untreated samples, as shown in Figure 2.

#### 2.3.1. Surface Treatment of Metallic Samples

Five metallic specimens were prepared by mechanical grinding using silicon carbide (SiC) abrasive papers with grit sizes ranging from 80 to 120 mesh, in order to homogenize and smooth the surface. Subsequently, a chemical cleaning process was performed by immersion in an acid solution composed of hydrofluoric acid (HF, 2.75 mol/L) and nitric acid (HNO_3_, 3.94 mol/L), in a 1:1 *v*/*v* ratio, for 2 min at room temperature, followed by rinsing with distilled water to remove reaction residues. After cleaning, the samples were subjected to an ultrasonic bath in distilled water for 30 min to ensure the complete removal of surface contaminants. To promote surface activation, the specimens were immersed in an oxidizing solution containing hydrogen peroxide (H_2_O_2_, 8.8 mol/L) and hydrochloric acid (HCl, 0.1 mol/L), also in a 1:1 *v*/*v* ratio, maintained at 80 °C for 30 min. Following this step, the samples were rinsed again with distilled water and subsequently underwent thermal treatment in a muffle furnace at 400 °C for 1 h, aiming to stabilize the surface and generate reactive sites favorable to HA nucleation.

#### 2.3.2. Surface Roughness Analysis

The surface roughness analysis was performed using a TIME roughness tester, model TR 200, with the purpose of evaluating the topographical changes resulting from the applied surface treatments. The instrument operates by tracing a stylus across the sample surface, where the vertical displacements of the probe needle are detected and converted into electrical signals. These signals are then processed by the internal system and displayed on the digital screen of the device. In this study, the arithmetic average roughness (Ra, μm) was measured for all Ti6Al4V alloy samples in order to quantify the degree of surface irregularity and correlate it with the respective preparation and treatment conditions.

#### 2.3.3. Deposition of HA by the Hydrothermal Method

The calcium oxide (CaO) powder obtained from the crab exoskeleton was used as a precursor for the synthesis of HA. The CaO powder was mixed with β-tricalcium phosphate (β-TCP; β-Ca_3_(PO_4_)_2_) and distilled water in a glass beaker, following the stoichiometric proportion required for the HA formation reaction, as expressed in Equation (1), adapted from [26]:

(1) $$3{Ca}_{3}{\left({PO}_{4}\right)}_{2}+CaO+H_{2}O\to {Ca}_{10}{\left({PO}_{4}\right)}_{6}{\left(OH\right)}_{2}$$

The quantities used were 0.72 g of calcium oxide, 11.62 g of tricalcium phosphate, and 30 g of distilled water. After preparing the reaction mixture, two sets of samples—CT (with surface treatment) and ST (without surface treatment), each containing five specimens—were placed in Petri dishes and covered with the prepared mixture. The magnetic stirring was maintained at 80 rpm, and the temperature was controlled at 37 °C, according to the parameters described by [26]. The heating process continued until the complete evaporation of the liquid, leaving only the solid residue adhered to the surface of the samples, which occurred after approximately 2 h for each group.

Although the methodology adopted in this study was adapted from the work of Hussain and Sabiruddin [27], it does not correspond to a conventional hydrothermal synthesis process, which typically requires high temperatures (≥200 °C) and elevated pressures in sealed autoclaves to promote crystallization under supercritical water conditions. Instead, the present route is a low-temperature aqueous precipitation process assisted by mild heating (≈37 °C), followed by in situ drying of the suspension on the metallic substrate. Therefore, it should be described more precisely as a thermally assisted wet-chemical deposition rather than a true hydrothermal method. This adaptation was intentionally implemented to avoid the use of high-pressure equipment and to maintain the process environmentally and economically accessible. Under these conditions, HA nuclei formed by reaction between β-Ca_3_(PO_4_)_2_ and CaO progressively aggregate and deposit on the substrate surface, resulting in a continuous porous coating after solvent evaporation.

Furthermore, it is acknowledged that the layer thickness values reported (95–98 µm) represent the apparent total coating thickness, including loosely bound agglomerates accumulated at the surface edges during drying, rather than a dense uniform film of that thickness. The effective continuous layer in the central region is estimated to be on the order of 5–10 µm, consistent with physical limitations of solvent evaporation methods.

### 2.4. Characterization of the Deposited HA Layer

#### 2.4.1. Optical Microscopy

A preliminary morphological analysis of the HA coating deposited on the ten metallic samples was performed using stereoscopic optical microscopy, aiming to visually inspect the uniformity, adhesion, and distribution of the ceramic layer on the substrates. The observations were carried out using a Stemi 508 Stereo Microscope (Carl Zeiss), located at the Inspection Laboratory of the Mechanical Engineering Technical Program, Federal Institute of Pará (IFPA)—Belém Campus.

In addition to the surface inspection, the average thickness of the HA layer was determined by optical microscopy of cross-sectioned samples. The coated specimens were embedded in phenolic resin, sectioned perpendicular to the coating surface, and carefully polished with SiC papers up to 1200 grit. The layer–substrate interface was then observed under reflected light using the same microscope, and measurements were taken at five distinct points along the section using the integrated image analysis software (AxioVision, Carl Zeiss).

#### 2.4.2. X-Ray Fluorescence Spectrometry (XRF)

To perform a qualitative analysis of the chemical composition of the deposited HA layer, an X-Ray Fluorescence (XRF) Spectrometer from the Materials Characterization Laboratory of the Metallurgy Technical Program, Federal Institute of Pará (IFPA)—Belém Campus, was employed. In order to avoid interference from the metallic substrate and ensure higher accuracy in the spectral readings, the deposited coating was carefully removed from the specimens, allowing the analysis to be carried out exclusively on the ceramic powder obtained after deposition.

#### 2.4.3. Scanning Electron Microscopy and Energy-Dispersive X-Ray Spectroscopy (SEM/EDS)

The morphological and chemical characterization of the HA coatings was carried out using Scanning Electron Microscopy (SEM) coupled with Energy-Dispersive X-ray Spectroscopy (EDS). The analyses were performed with a Vega 3 Scanning Electron Microscope (TESCAN), located at the Metallography Laboratory of the Materials Engineering Program, Federal Institute of Pará (IFPA)—Belém Campus. Secondary electron micrographs were obtained under the following operating conditions: beam current of 90 μA, constant accelerating voltage of 20 kV, and working distance of 15 mm. Images were captured at magnifications of 1000×, 5000×, 20,000×, and 30,000×, enabling detailed observation of the surface morphology and distribution of the deposited particles. For the EDS chemical analysis, in order to minimize interference and ensure higher accuracy in the quantification of elemental composition, the analysis was performed on powder samples obtained without immersion of the metallic substrates, thereby simulating measurements exclusively on the deposited ceramic layer.

#### 2.4.4. X-Ray Diffraction (XRD)

X-Ray Diffraction (XRD) analysis was employed to identify and characterize the crystalline phases present in the ceramic material deposited on the metallic substrates. The measurements were carried out at the Materials Characterization Laboratory of the Institute of Geosciences, Federal University of Pará (UFPA), using an Empyrean X-Ray Diffractometer (PANalytical) equipped with a ceramic X-ray tube with a cobalt (Co) anode and Kα_1_ radiation (λ = 1.789010 Å), a long fine focus, and an iron (Fe) Kβ filter. The analyses were performed in scanning mode, under an accelerating voltage of 40 kV and a current of 35 mA, using a PIXCEL3D–Medipix3 1 × 1 detector. The angular scan was conducted from 2° to 75° (2θ), with a step size of 0.026° (2θ) and a counting time of 31 s per step. The instrumental geometry included a divergence slit of 1/4°, an anti-scatter slit of 1/2°, and a 10 mm mask. The diffraction data obtained were processed and analyzed using the X’Pert HighScore Plus software with the PDF-2021 database, enabling phase identification and qualitative structural analysis of the HA coating.

### 2.5. Statistical Treatment and Sample Size

All experimental results were obtained from multiple independent specimens to ensure statistical reliability. Chemical composition was determined on two samples with four sequential burns each, and hardness values were obtained from two specimens with ten indentations per sample. Surface roughness (Ra) measurements were performed in triplicate on five treated (CT) and five untreated (ST) specimens, while coating thickness was measured at five distinct points along each cross-sectioned sample. For elemental analyses (EDS and XRF), at least two replicate scans were acquired per sample. The data were processed using standard descriptive statistics (mean, standard deviation, and standard error). When appropriate, analysis of variance (ANOVA) was applied at a 99.7% confidence level to verify the statistical consistency and homogeneity of the results. All quantitative results are reported as mean ± standard deviation unless otherwise stated.

## 3. Results and Discussion

### 3.1. Characterization of Metallic Material

#### 3.1.1. Chemical Composition

Table 1 presents a comparison between the chemical composition of the titanium substrates used in this study and the values specified for the commercial Ti-6Al-4V alloy according to the ASTM F136-18 [28].

Based on the average results obtained by optical emission spectrometry (OES), the analyzed samples contained 88.75 wt.% Ti, 7.46 wt.% Al, and 3.41 wt.% V. When compared to the standardized composition range (Al = 5.5–6.5 wt.%; V = 3.5–4.5 wt.%; Ti = balance), these results reveal a deviation from the typical Ti-6Al-4V alloy. The aluminum content exceeds the upper limit specified by the standard, while the vanadium content is slightly below the minimum value. Therefore, the alloy used in this study can be classified as a non-conforming variant of Ti-6Al-4V and should be referred to as a Ti-Al-V alloy outside the ASTM F136-18 [28].

These compositional deviations have direct implications for the alloy’s microstructure and mechanical behavior. The excess aluminum, an α-phase stabilizer, combined with the slight deficiency of vanadium, a β-phase stabilizer, indicates a tendency toward a higher fraction of the α phase and a corresponding reduction in the β phase at room temperature. This shift may lead to an alloy exhibiting a slightly higher elastic modulus and strength, but with reduced ductility and toughness, as well as an increase in the β-transus temperature.

In the context of HA deposition, such compositional differences may influence the surface chemistry of the substrate. The higher aluminum content promotes the formation of aluminum oxides (Al_2_O_3_) rather than vanadium oxides, thereby modifying surface energy, wettability, and local charge distribution—factors that can affect the nucleation and adhesion behavior of the HA coating.

The observed deviations may arise from several factors, including the use of a titanium alloy batch that does not fully comply with Ti-6Al-4V specifications, surface segregation phenomena (alpha-case) induced by thermal oxidation or processing, or analytical uncertainties inherent to the OES method, such as matrix calibration, burn depth, and argon purity during purging.

Thus, the critical assessment of the results indicates that the metallic substrates employed in this study do not fully correspond to the standardized Ti-6Al-4V alloy but rather to a compositional variation of it. This distinction must be considered in the interpretation of microstructural and mechanical results, as well as in the evaluation of the interfacial behavior between the titanium substrate and the deposited HA layer.

Therefore, throughout the manuscript we refer to the substrate as a Ti–Al–V α + β alloy outside the ASTM F136-18 [28] limits, while noting that its hardness and microstructure remain typical of α-rich Ti–6Al–4V-like materials.

#### 3.1.2. Optical Microscopy

**Figure 3:**
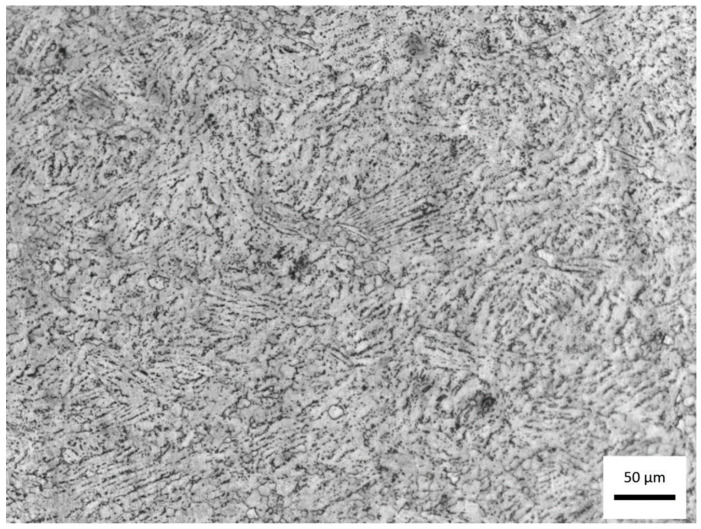
Micrograph obtained by optical microscopy with 200× magnification, after chemical attack by immersion in Kroll reagent for 80 s, showing the morphology and distribution of the microstructural phases of the material.

Figure 3 shows the micrograph of the titanium alloy studied, obtained by optical microscopy at 200× magnification after chemical etching by immersion in Kroll’s reagent for 80 s.

The observed morphology reveals a typical α + β titanium alloy microstructure, although certain features suggest a chemical composition slightly outside the standard range of Ti-6Al-4V, as previously discussed. The image displays a matrix predominantly composed of the α phase (hexagonal close-packed), visible as light, elongated, and acicular regions, interspersed with thin, dark areas corresponding to the β phase (body-centered cubic). This structural arrangement is characteristic of α + β alloys air-cooled from the β region, indicating solidification and subsequent heat treatment without controlled aging.

The irregular distribution and fine lamellar morphology of α within the β matrix suggest an intermediate cooling rate, consistent with hot-worked alloys cooled in air, with no evidence of martensitic α′ formation. The absence of well-defined α colonies and the presence of fine, dispersed lamellae can be attributed to the higher aluminum content (7.46 wt.%) identified in the chemical analysis, which acts as an α-phase stabilizer, raising the β-transus temperature and promoting microstructural refinement. Conversely, the slightly lower vanadium content (3.41 wt.%), a β-phase stabilizer, contributes to a reduction in the β phase fraction, supporting the predominance of α observed in the micrograph.

The contrast achieved through Kroll’s reagent etching clearly delineates the α grain boundaries and the interlamellar β phase, confirming a typical α + β morphology with a dominant α phase. This microstructural configuration generally results in higher mechanical strength and elastic modulus, albeit at the expense of ductility and fracture toughness, as expected for aluminum-enriched α + β titanium alloys.

Overall, the micrograph confirms that the material retains the α + β morphology characteristic of Ti-Al-V alloys, but with a strong tendency toward α-phase stabilization, consistent with the chemical composition deviations identified by spectrometric analysis. This predominance of the α phase may directly influence the surface behavior during HA deposition, since the topography and oxide chemistry are strongly dependent on the crystallographic nature of the exposed phase.

**Figure 4:**
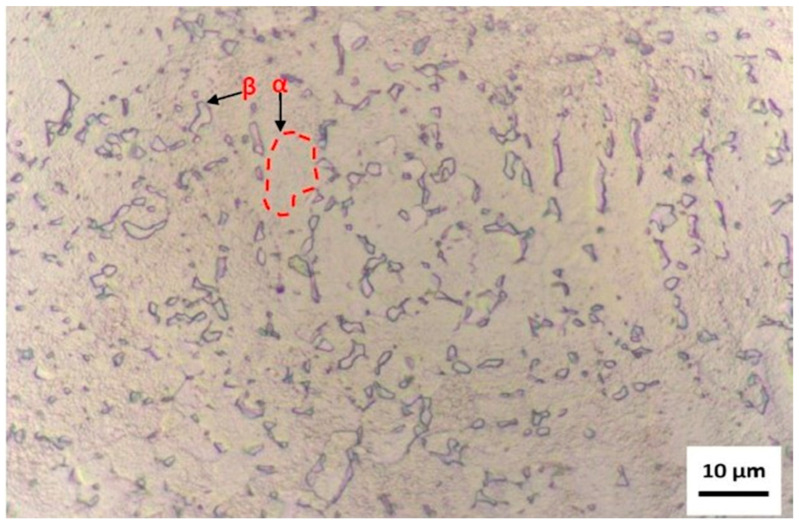
Micrograph obtained by optical microscopy at 1000× magnification, after chemical etching by immersion in Kroll’s reagent for 80 s. The α and β phases of the alloy can be observed, with emphasis on the morphology and distribution of the α phase regions (in red).

Figure 4 presents the micrograph of the titanium alloy studied, obtained by optical microscopy at 1000× magnification after chemical etching in Kroll’s reagent for 80 s, followed by image enhancement for phase distinction.

The microstructure reveals a dual-phase morphology (α + β) typical of titanium alloys with compositions near Ti-6Al-4V; however, the relative phase proportions and morphology indicate a predominance of the α phase, consistent with the chemical analysis results, which identified an aluminum content above and a vanadium content slightly below the ASTM F136-18 [28] specification limits.

The α phase (hexagonal close-packed) appears as elongated or equiaxed light regions, while the β phase (body-centered cubic) is observed as dark intergranular regions, often forming thin, discontinuous networks. This phase distribution suggests a non-homogeneous transformation from β to α during cooling, indicative of an intermediate cooling rate typical of air-cooled or furnace-cooled samples after hot working. The discontinuous nature of the β boundaries and the fine dispersion of α grains point to a microstructure refined by the higher aluminum content (7.46 wt.%), which increases the β-transus temperature and promotes α-phase stabilization.

The volume fraction of the β phase appears reduced, which corroborates the slightly lower vanadium content (3.41 wt.%) found in the chemical composition analysis. Vanadium is a β-stabilizing element; therefore, its deficiency results in a microstructure biased toward α-phase dominance. The grain morphology also indicates a lamellar α within prior β grains, characteristic of a Widmanstätten or basket-weave microstructure, which often forms when the alloy is cooled moderately from the β region. Such a microstructure tends to enhance strength and hardness, but may reduce ductility and fracture toughness—a balance consistent with the mechanical performance typically observed in α-rich titanium alloys.

The contrast obtained through Kroll’s reagent effectively delineates the grain boundaries and interphase regions, allowing clear visualization of the α/β interfaces. The uniform etching response and the absence of secondary precipitates suggest a homogeneous distribution of alloying elements, though local differences in contrast might also indicate slight chemical segregation at α/β interfaces or incipient oxidation from surface exposure prior to etching.

From a metallurgical perspective, this α-dominant α + β microstructure reflects the compositional deviations previously identified and confirms that the alloy, while closely resembling Ti-6Al-4V, corresponds to a non-standard Ti-Al-V composition with a greater α-phase stability. This microstructural configuration can influence the surface reactivity and oxide formation behavior during subsequent processing, particularly affecting HA nucleation and coating adhesion, as the α phase tends to form more stable TiO_2_ and Al_2_O_3_ oxides, altering surface charge and energy.

#### 3.1.3. Scanning Electron Microscopy (SEM) and X-Ray Dispersive Spectroscopy (EDS)

**Figure 5:**
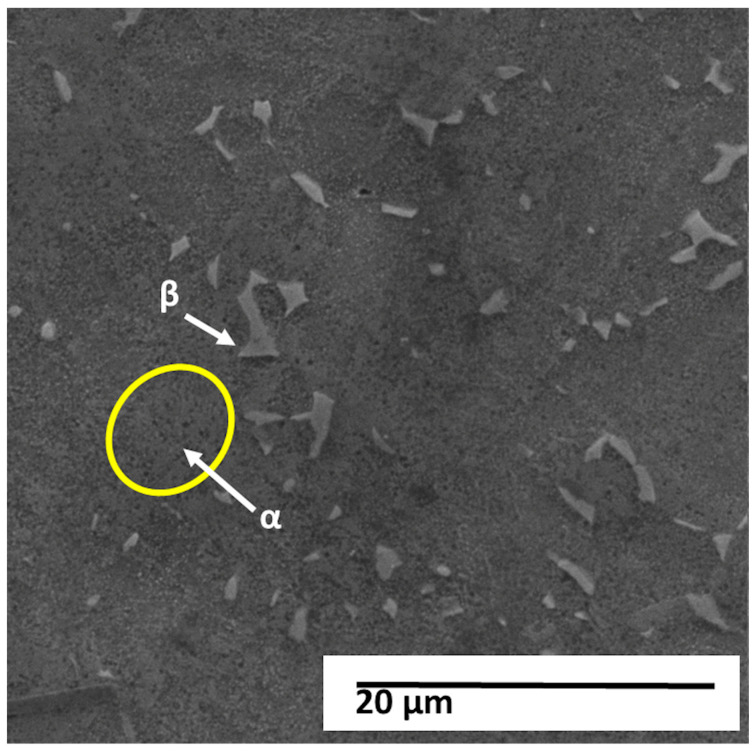
SEM micrograph of the Ti–Al–V alloy showing the α and β phases of the microstructure. The brighter regions correspond to the α phase, while the darker lamellae represent the β phase. Scale bar = 20 µm.

Figure 5 shows the micrograph obtained by scanning electron microscopy (SEM) with a magnification of 5000×, working distance (WD) of approximately 15.0 mm, accelerating voltage of 20 kV, and combined detection by secondary (SE) and backscattered electrons (BSE).

The image shows the surface of the titanium alloy under study, revealing a two-phase (α + β) microstructure with smooth surface relief and the presence of discontinuous, finely distributed lamellae with micrometer dimensions. The highlighted regions indicate the typical α and β phases of this alloy.

In secondary electron images, the darker, recessed regions generally correspond to the β phase (vanadium-enriched and body-centered cubic—BCC) while the lighter, more prominent regions represent the α phase (aluminum-stabilized and hexagonally close-packed—HC). The observed contrast is predominantly topographic, resulting from chemical attack and the difference in corrosion rates between the phases. The presence of short, fragmented, and slightly continuous β lamellae embedded in an α matrix reinforces the characteristic of a Widmanstätten (or “wicker basket”) microstructure, formed at intermediate cooling rates, typical of air cooling after hot processing.

The fine dispersion of the α phase and the discontinuity of the β phase are consistent with a chemical composition outside the typical range of the Ti-6Al-4V alloy, as previously identified. The higher aluminum content (7.46%) acts as a stabilizer of the α phase, raising the β transition temperature (β-transus) and promoting microstructural refinement. On the other hand, the lower vanadium content (3.41%), a stabilizer of the β phase, reduces the volume fraction of the β phase, resulting in a predominantly α matrix, which explains the appearance observed in the image.

The absence of α′ martensite and the presence of refined α lamellae indicate that cooling was not rapid enough to generate martensite, but also not slow enough to allow the growth of coarse colonies—typical behavior of hot-worked and air-cooled materials. Small pits and polishing flaws observed on the surface suggest artifacts of metallographic preparation, while isolated bright spots can be attributed to contamination residues or reprecipitation of material during etching or conductive coating.

The observed α-dominant microstructure tends to exhibit higher elastic modulus and mechanical strength, although with lower ductility and fracture toughness, compared to more β-rich alloys. This configuration is consistent with the measured chemical composition, confirming that the analyzed material is a Ti-Al-V alloy outside the ASTM F136-18 [28] specifications.

From a functional perspective, the predominance of the α phase directly influences the surface behavior of the material, especially regarding oxide formation. α surfaces tend to form more stable layers of TiO_2_ and Al_2_O_3_, which can alter the surface energy, wettability, and local electrical charge, affecting the nucleation and adhesion of the HA layer. The high density of α/β interfaces can, on the other hand, favor the heterogeneous nucleation of HA due to the increased number of active sites and crystalline defects.

**Figure 6:**
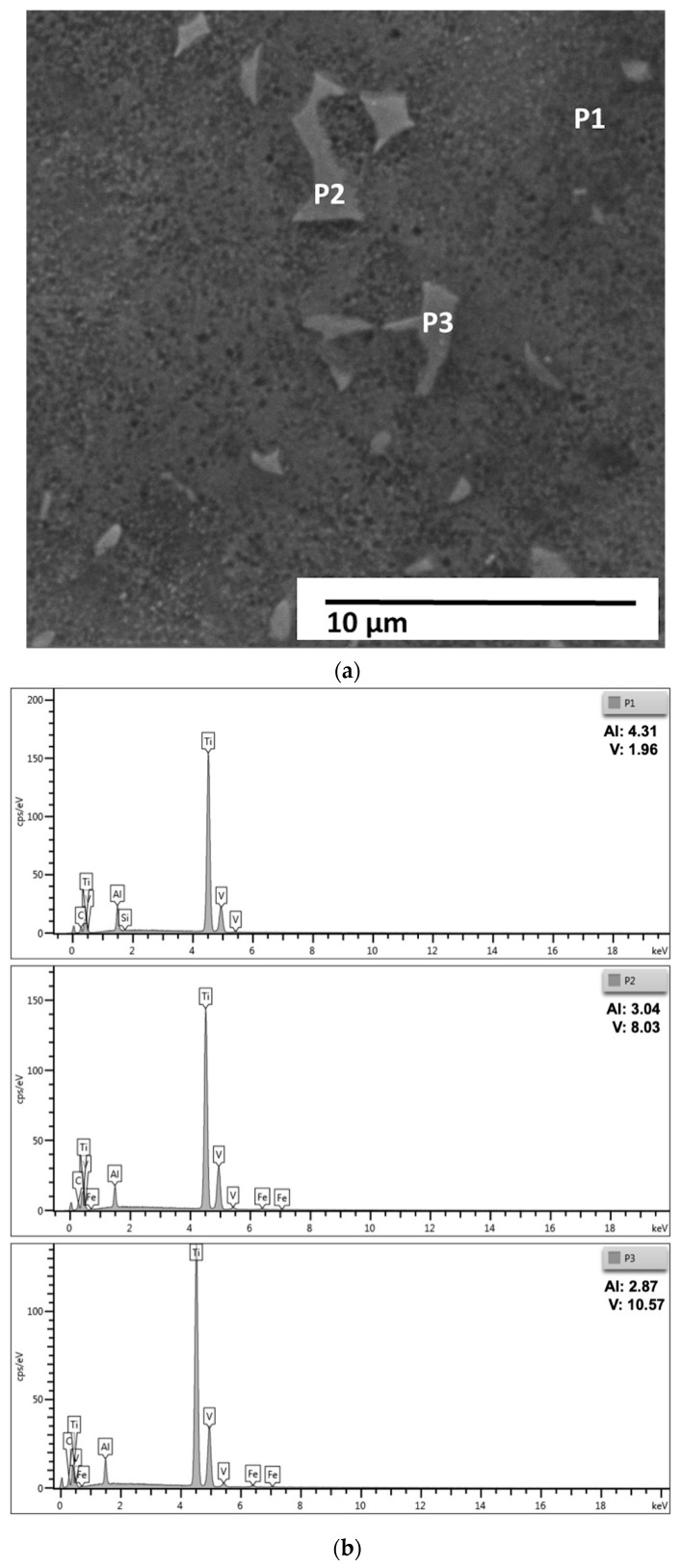
(**a**) SEM micrograph of the Ti–Al–V alloy showing the lamellar distribution of the α and β phases at higher magnification. (**b**) Enlarged SEM micrograph of the same region, including an annotated inset highlighting the EDS analysis points (P1, P2, and P3) and local microstructural features. Scale bar = 10 µm.

A more detailed view of the same region, shown in Figure 6, reveals the fine lamellar arrangement of α and β phases and identifies the EDS points selected for local compositional analysis. Figure 6 shows the micrograph obtained by scanning electron microscopy (SEM), with a magnification of 10,000× and an acceleration voltage of 20 kV, in which three points of punctual analysis by energy dispersive X-ray spectroscopy (EDS) were identified, called P1, P2 and P3.

The spectra obtained reveal significant differences in the concentrations of aluminum (Al) and vanadium (V), reflecting the chemical heterogeneity typical of two-phase titanium alloys. At point P1, an Al content of 4.31% and V of 1.96% were observed, while at points P2 and P3 the measured values were, respectively, Al = 3.04%; V = 8.03% and Al = 2.87%; V = 10.57%.

This local compositional variation is consistent with the partitioning behavior of alloying elements between the α (hexagonal close-packed) and β (body-centered cubic) phases in Ti-Al-V alloys. Point P1, which has a higher aluminum concentration and lower vanadium content, can be associated with the α phase, stabilized by Al. Points P2 and P3, with higher vanadium and lower aluminum contents, are characteristic of the β phase, stabilized by V. Thus, the EDS results corroborate the presence of an α + β microstructure, in which there is a predominance of the α phase and discontinuous regions of β, in agreement with the previous micrographs and with the global chemical analysis obtained by optical emission spectrometry (OES).

The average chemical composition obtained by OES (Al = 7.46%; V = 3.41%) indicated an alloy outside the Ti-6Al-4V composition range standardized by ASTM F136-18 [28], presenting excess Al and V deficiency. The specific EDS results, therefore, confirm the expected partitioning: regions with local Al enrichment associated with the α phase and V-enriched regions corresponding to the β phase. This microchemical heterogeneity is typical of α + β alloys and explains the differences between local measurements and the average material composition, since EDS evaluates micrometric volumes and integrates information from different lamellae and interfaces.

Secondary Si and Fe peaks were also observed in the spectra, likely resulting from surface contamination from the metallographic preparation process, such as silica-based abrasive residues, cutting disc particles, or metal fragments from equipment. These elements appear in very low concentrations and do not indicate the presence of significant intermetallic phases.

Morphological analysis, combined with EDS data, reinforces the conclusion that the material exhibits a refined Widmanstätten microstructure, consisting of finely distributed α lamellae and discontinuous β regions, characteristic of intermediate cooling after thermomechanical processing. This configuration results from a higher Al content, which raises the β transition temperature (β-transus) and promotes a higher α-phase fraction, and a lower V content, which reduces β-phase stability.

From a material properties perspective, the predominance of the α phase tends to provide higher elastic modulus and mechanical strength, but lower ductility and fracture toughness, compared to alloys with a higher β fraction. This characteristic should be considered when interpreting mechanical tests and analyzing surface behavior during the HA deposition process. Local differences in the distribution of the α and β phases also affect oxide layer formation, influencing wettability, surface energy, and surface electrical charge, which can modify HA nucleation and adhesion.

#### 3.1.4. Hardness

**Figure 7:**
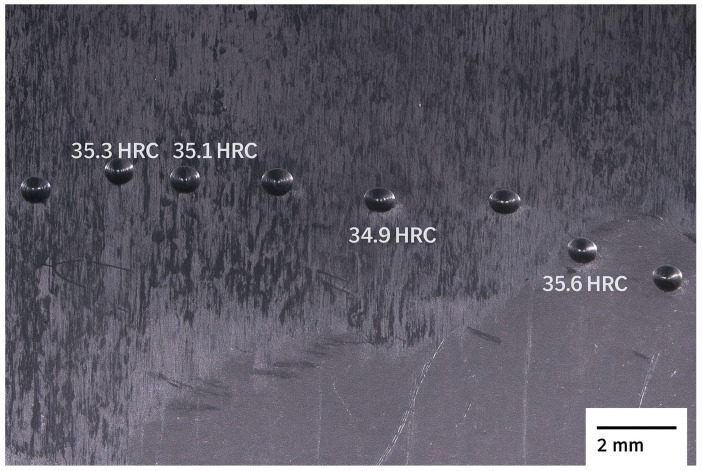
Optical image of the Rockwell C hardness test region on the Ti–Al–V substrate. Four representative hardness values (in HRC) were added next to selected indentations. Scale bar = 2 mm.

Figure 7 shows the indentations obtained during the Rockwell hardness test, in which ten indentations were made per sample, arranged in a centerline and at random positions, to determine the average hardness of the material representatively, without concentrating the measurements on specific regions. For clarity, the individual Rockwell C hardness values obtained at each test point have been added directly to Figure 7, positioned adjacent to the corresponding indentations. This experimental strategy is appropriate for α + β titanium alloys, whose microstructure presents intrinsic heterogeneity due to the simultaneous presence of α lamellae and β regions, preventing the reading from being influenced by local phase variations.

The image shows that the indentations are well-defined and distributed throughout the sample, indicating a uniform test. However, sanding marks and residual surface scratches are noticeable, suggesting that the surface finish could be improved to reduce roughness and, consequently, minimize reading errors. Excessively rough or poorly polished surfaces can cause artificial variations in the measurement, as the indenter may impact micro-elevations or depressions, generating slightly higher or lower hardness values than the actual ones. Therefore, it is recommended that sample preparation include fine sanding (up to 1200 grit) followed by light polishing, ensuring homogeneity and the absence of metal debris under the indenter.

Table 2 presents the average Rockwell C hardness (HRC) values obtained for samples A1 and A2 of the studied titanium alloy, as well as the reference values available in the ASM Handbook, Volume 2 [29], for the Ti-6Al-4V alloy in as-supplied condition.

The hardness measurements obtained for the Ti–Al–V alloy showed good consistency between samples and fell within the range expected for Ti–6Al–4V–type alloys in the as-received condition. Both specimens exhibited low variability between indentations, indicating microstructural uniformity at the local scale. The values are also compatible with an α-rich α + β microstructure, as observed in the metallographic analysis, which typically leads to hardness values slightly above those of alloys with higher β content. These findings confirm that, although the chemical composition deviates slightly from ASTM F136-18 [28] limits, the mechanical response remains characteristic of conventional Ti–6Al–4V alloys.

Statistical analysis shows that both samples presented low dispersion of results, with standard deviations of 0.499 HRC for A1 and 0.923 HRC for A2, and corresponding standard errors of 0.157 HRC and 0.292 HRC, respectively. Sample A2 presented slightly greater variation, which may be associated with the microstructural heterogeneity observed in the metallographic analyses, since small local differences in the fraction of α and β phases can affect the response to the hardness test. Furthermore, subtle differences in surface preparation, effective sample thickness, or the distance between indentations may also have contributed to the greater variability observed in A2.

Considering the 95% confidence interval, the means obtained for A1 (35.07 ± 0.31 HRC) and A2 (35.83 ± 0.57 HRC) overlap the range established by ASM, confirming that the measured values are statistically compatible with the expected range for Ti-6Al-4V alloys. The difference between the means of the two samples (0.76 HRC) is small and, although statistically detectable, does not represent a practically significant difference. This variation can be attributed to small local fluctuations in the microstructure—regions richer in the α phase, stabilized by aluminum, tend to present greater hardness, while areas with a higher fraction of the β phase, stabilized by vanadium, exhibit slightly lower values.

The hardness results are consistent with the microstructural and chemical characterization discussed previously. The micrograph showed an α + β structure with a predominance of α, which is consistent with the presence of a higher aluminum and lower vanadium content compared to the standard Ti-6Al-4V alloy. This microstructural configuration tends to slightly increase the hardness and elastic modulus of the material, although it may reduce its ductility and fracture toughness. Thus, the hardness values obtained confirm the expected mechanical behavior for an α-dominant Ti-Al-V alloy, corroborating the conclusions drawn from the metallographic and chemical analyses.

Table 3 shows the arithmetic roughness averages (Ra) measured in triplicate on untreated surfaces (ST) and on surfaces subjected to sanding + thermochemical treatment (CT).

The untreated surfaces (ST) showed a markedly heterogeneous roughness profile, suggesting significant variability in the starting surface condition, likely stemming from prior machining marks and oxidation differences. In contrast, the treated surfaces (CT) exhibited a highly uniform roughness, indicating that the thermochemical protocol not only reduced variability but also standardized the surface topography. This homogenization is beneficial for coating processes, as more uniform surfaces tend to promote consistent HA nucleation and adhesion. Additionally, the slight decrease in mean Ra after treatment suggests improved wettability and surface reactivity, which align with the enhanced coating uniformity observed in later analyses.

The intra-sample standard deviations (“D. Standard” lines) are low in both groups (typically 0.008–0.059 µm in ST and 0.010–0.017 µm in CT), indicating good profilometer repeatability in the same region. However, the inter-sample variability is clearly higher in ST (due to the wide range of means), while in CT it practically disappears (all means ~0.587 µm). The listed standard errors (S/√3) confirm the metrological consistency of the readings.

From a functional point of view, the Ra range of ~0.55–0.60 μm obtained after the treatment is suitable for HA nucleation and adhesion, as several studies report that surface roughness values between approximately 0.2 and 1.5 μm promote ion adsorption, early-stage nucleation, and improved bonding of Ca–P phases on titanium substrates [3,6]. It is also noted that the treatment acted as a “normalizer”: on very smooth surfaces (e.g., A4–ST = 0.416 µm), Ra increased to ~0.587 µm; on very rough surfaces (e.g., A2–ST = 0.954 µm), Ra decreased to the same level. This convergence tends to standardize surface energy and wettability, favoring more homogeneous coatings.

The thermochemical treatment not only homogenized the roughness but also modified the surface chemistry through oxidation and hydroxylation, increasing surface energy and wettability. These effects favor the initial adsorption of Ca^2+^ and PO_4_^3−^ ions from the suspension and facilitate heterogeneous nucleation of HA, which explains the improved coating continuity observed later by optical and electron microscopy. The resulting Ra ≈ 0.587 µm lies within the optimal range reported for HA nucleation on titanium (0.2–1.0 µm), ensuring sufficient anchoring without compromising coating uniformity.

#### 3.1.5. Correlation Between Substrate Features and Coating Behavior

The chemical and microstructural features of the Ti–Al–V substrate directly affect the adhesion and growth of the HA layer. The predominance of the α phase and the presence of α/β interfaces increase the density of high-energy sites and crystallographic mismatches that act as preferential nucleation points for Ca–P compounds. Likewise, the uniform hardness (~35 HRC) and fine Widmanstätten morphology provide a mechanically stable base that resists plastic deformation during coating formation and drying. These characteristics are expected to enhance interfacial bonding by combining mechanical interlocking with localized chemical reactivity. Therefore, understanding the substrate state is essential to interpret the coating morphology and the effect of thermochemical treatment discussed in the following sections.

### 3.2. Characterization of the Deposited HA Layer

#### 3.2.1. Optical Microscopy

**Figure 8:**
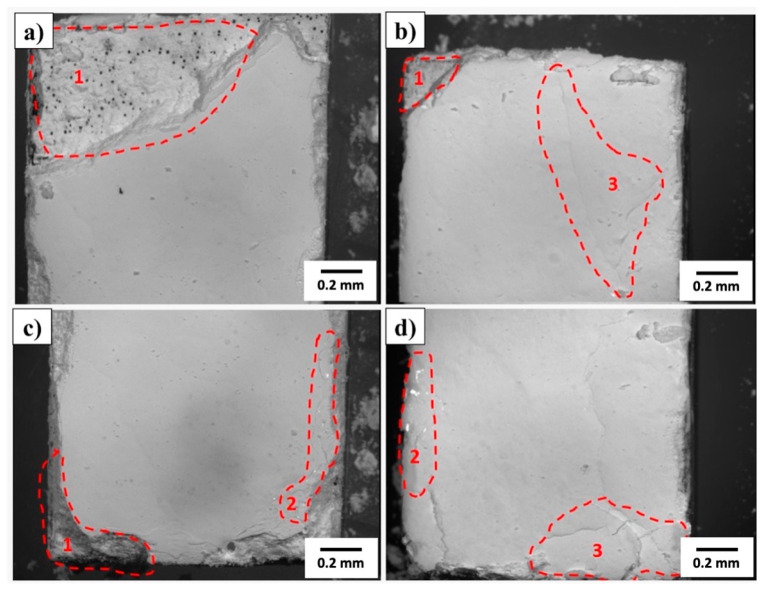
Optical microscopy of HA-coated surfaces after drying (24 h in a silica desiccator): (**a**,**b**) specimens without surface treatment (ST); (**c**,**d**) specimens with thermochemical treatment (CT). The hatched areas highlight typical morphological variations (peripheral accumulation/meniscus and shrinkage microcracks). Scale bar: 0.2 mm.

Figure 8 shows, by optical microscopy, the surface morphology of the HA layers obtained after drying the samples in a desiccator for 24 h. Quantitative surface roughness measurements were not performed after the HA deposition. However, qualitative analysis based on optical and electron micrographs indicates that the coating increased the apparent surface roughness relative to the polished titanium substrate. The formation of porous, granular, and acicular HA agglomerates produced a micro–nano hierarchical topography, visually more irregular than the underlying metal surface (Ra ≈ 0.587 µm). Therefore, although the mean Ra value after coating was not measured, it can be inferred that the overall surface roughness increased due to the intrinsic porosity and crystallite morphology of the deposited HA layer.

In all samples, complete coverage of the metal substrate is observed, with an opaque white color and a plaster-like appearance, consistent with a water-deposited ceramic film. However, detailed analysis of the images reveals the presence of local heterogeneities that deserve attention regarding the thickness, continuity, and adhesion of the deposited layer.

In specific regions of the micrographs, labeled 1, 2, and 3, material accumulates at the edges and corners of the samples, forming a thicker layer, characteristic of drying by meniscus recession, a phenomenon known as coffee-ring. This effect occurs due to the capillary migration of solid particles from the center to the edges during solvent evaporation, resulting in a thickness gradient between the center and the edges of the sample. Consequently, thicker edges tend to concentrate residual drying stresses, increasing the propensity for microcrack formation and localized spalling.

Fine cracks and shrinkage marks are also visible on the film surface, particularly in the areas labeled 3. These discontinuities are attributed to the volumetric contraction of the ceramic matrix during the drying process, typical of aqueous suspensions with a high solids fraction. This shrinkage is intensified by the differential adhesion between the film and the substrate, as surfaces with better anchoring impose greater internal traction on the film. Additionally, small gaps and isolated pores can be observed, related to the trapping of air bubbles, non-dispersed agglomerates, or particles detached during drying. Although uncommon, these defects act as stress concentrators and can serve as initiation points for mechanical failure or debonding.

Table 4 presents the thickness measurements of the HA layers deposited on the metal substrates with and without surface treatment, expressed in micrometers (µm).

In general, the average thicknesses obtained for the untreated (ST) and treated (CT) specimens are similar—95 µm and 98 µm, respectively—indicating that, in terms of overall average thickness, the thermochemical treatment did not cause a significant variation in the amount of deposited material. However, individual analysis of the samples reveals distinct behaviors and marked heterogeneity among the specimens, demonstrating that the surface treatment influenced deposition inconsistently.

In the ST group, thicknesses ranged from 80 to 129 µm, resulting in a range of 49 µm and a coefficient of variation of approximately 25%, indicating considerable dispersion among the samples. In the CT group, the variation was even more pronounced, with values between 57 and 135 µm and a range of 78 µm, corresponding to a coefficient of variation exceeding 40%. These results indicate that the thermochemical treatment did not bring uniformity to the layer thickness; on the contrary, it appears to have increased thickness variability.

Analyzing the samples individually, it is clear that the effect of the treatment was not systematic. In two of them (Samples 01 and 03), the thickness decreased after treatment, going from 90 to 57 µm and from 80 to 71 µm, respectively. In contrast, in Samples 02 and 04, the thickness increased considerably, reaching 131 µm and 135 µm, compared to 80 µm and 129 µm in the untreated samples. This random variation between increase and decrease suggests that local and procedural factors, such as wettability, surface tension, suspension rheology, and drying conditions, exerted a greater influence on the result than the chemical treatment itself.

These findings are consistent with observations made using optical microscopy (Figure 8), which revealed the presence of material accumulations at the edges (coffee-ring effect), shrinkage microcracks, and center-to-edge thickness gradients, typical of ceramic films formed by aqueous deposition and drying. The non-uniformity in material distribution can be explained by capillary migration of solid particles during evaporation, which leads to peripheral thickening and the formation of thinner zones in the center of the sample. Furthermore, small differences in surface energy between the treated and untreated groups may have modified the wettability of the HA suspension, resulting in thinner or thicker layers, depending on the balance between spreading and adhesion.

Another important aspect is the possible influence of the measurement point. If measurements were taken in different regions (edge, center, or intermediate areas), it is natural that the differences will be accentuated, especially in films with a thickness gradient. Thus, the observed variability may reflect not only the actual morphology of the film, but also local variations in the measurement methodology.

Overall, the heterogeneous layer thicknesses observed in both groups can be interpreted by considering surface energy and liquid-film dynamics during drying. Treated samples, despite exhibiting similar mean Ra values, have higher surface polarity due to the formation of TiO_2_ and Al_2_O_3_–OH groups. This chemical activation promotes stronger local adhesion but also induces differential capillary flows during solvent evaporation, leading to peripheral thickening (coffee-ring effect) and microcracking. Hence, the surface treatment improves wetting and adhesion at the expense of slightly higher morphological heterogeneity.

Although no direct mechanical testing of the HA coating was performed, qualitative observations indicate that the deposited layer exhibited good adherence and cohesion. The coating remained firmly attached to the Ti–Al–V substrate during handling, sectioning, and polishing for cross-sectional microscopy, showing no signs of delamination or detachment. This behavior suggests sufficient interfacial bonding for laboratory-scale evaluation. However, the mechanical strength and long-term durability of the coating under physiological or mechanical stress conditions were not quantified in this study. Future work will include adhesion tests (e.g., scratch or pull-off methods) and immersion durability assessments in simulated body fluid (SBF) to evaluate the coating’s mechanical stability and its evolution over time. Such analyses will be essential to confirm the practical applicability of the biogenic HA layer for biomedical use.

It should be noted that quantitative adhesion testing was not performed in this study. Only qualitative assessment was conducted during handling, cross-sectioning, and polishing, where the HA layer remained attached to the substrate without signs of delamination. Although this confirms adequate cohesion for laboratory-scale manipulation, it does not substitute for standardized mechanical adhesion tests. ISO 13779-2:2018 [30] establishes that HA coatings intended for high biomechanical loads must exhibit tensile bond strength values ≥15 MPa. Future work will therefore include quantitative adhesion measurements (e.g., pull-off or scratch testing) to determine compliance with ISO requirements and to more rigorously assess the coating’s suitability for biomedical implant applications.

#### 3.2.2. X-Ray Fluorescence (XRF)

Although the XRD pattern of the calcined precursor was not obtained, XRF analysis was performed to confirm the presence of calcium oxide as the major constituent. As shown in Table 5, CaO is the predominant oxide, followed by P_2_O_5_, with only trace contributions from other oxides such as SrO and K_2_O. This composition indicates that the material is markedly calcium-rich, a characteristic frequently observed in apatites derived from marine sources.

The Ca-enriched profile implies that the conversion of CaCO_3_ → CaO → HA was not fully stoichiometric, and that a fraction of CaO or Ca(OH)_2_ likely remained unreacted during wet-chemical synthesis. This effect is expected in low-temperature routes where diffusion and reaction kinetics limit complete phase transformation. Consequently, the calculated Ca/P ratio derived from the oxide proportions is higher than the stoichiometric value of HA (1.67), suggesting the coexistence of secondary Ca-based phases rather than a single-phase apatitic material.

From a functional perspective, such Ca-rich compositions may increase initial alkalinity, solubility, and ionic release—parameters that typically accelerate early-stage bioactivity and the formation of secondary apatite in physiological media. However, excessive CaO/Ca(OH)_2_ content may also influence coating stability or induce hydration-related microcracking. Therefore, the XRF findings are consistent with the morphological and EDS observations, which also highlighted slight Ca enrichment relative to phosphorus.

#### 3.2.3. Scanning Electron Microscopy (SEM) and X-Ray Dispersive Spectroscopy (EDS)

**Figure 9:**
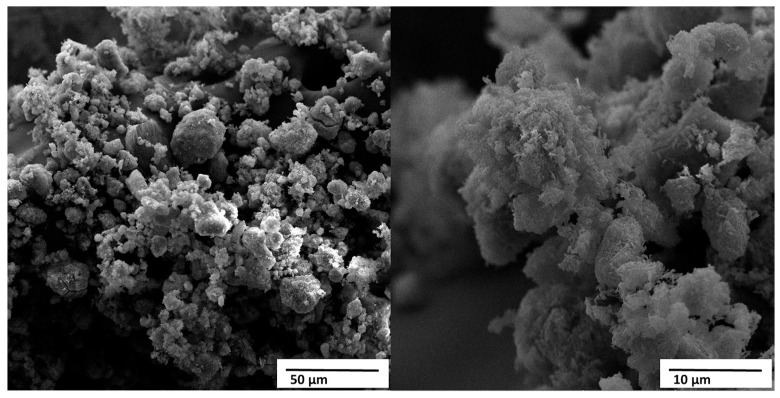
SEM micrographs of the crab shell–derived HA (HACRAB) powder showing agglomerated porous structures at two magnifications. Scale bars = 50 µm (**left**) and 10 µm (**right**).

Figure 9 shows the micrographs obtained by scanning electron microscopy (SEM) of the HA powder derived from crab shell, called HACRAB, under magnifications of 1000× and 5000×.

At lower magnification, the material displays heterogeneous aggregates ranging from approximately 10 to 80 μm, formed by the association of smaller submicrometric particles. At higher magnification, these agglomerates are seen to consist of fine granular or nodular crystallites with sizes typically between 0.1 and 1 μm. Such multi-scale porosity and hierarchical structuring are characteristic of apatites synthesized from marine biowaste and are frequently associated with enhanced ionic exchange and accelerated reactivity in physiological environments [10,11].

The presence of partially fused grains and locally densified regions suggests that some thermal coalescence occurred during the calcination of the precursor shells at 800 °C. These features are consistent with the high CaO content identified by XRF (Table 5), which indicates that the conversion to hydroxyapatite was not entirely stoichiometric. In Ca-rich systems, residual CaO or Ca(OH)_2_ phases often remain distributed within the apatite agglomerates, contributing to the formation of denser spots among otherwise porous structures. This Ca enrichment also explains the higher-than-stoichiometric Ca/P ratio and is known to increase early-stage solubility and ion release, both of which can promote rapid secondary apatite precipitation in simulated body fluids [10].

The overall texture observed in Figure 9, porous agglomerates composed of fine submicrometric crystallites, is typical of biogenic HA obtained through wet-chemical routes and correlates well with the material’s expected bioactivity. Highly porous microstructures increase the specific surface area, facilitate protein adsorption, support heterogeneous nucleation of new apatite layers, and enhance osteoconductivity. These characteristics have been widely reported for nanostructured or biogenically derived calcium phosphates, which often exhibit improved biological affinity compared to dense, high-temperature synthetic HA powders [13].

**Figure 10:**
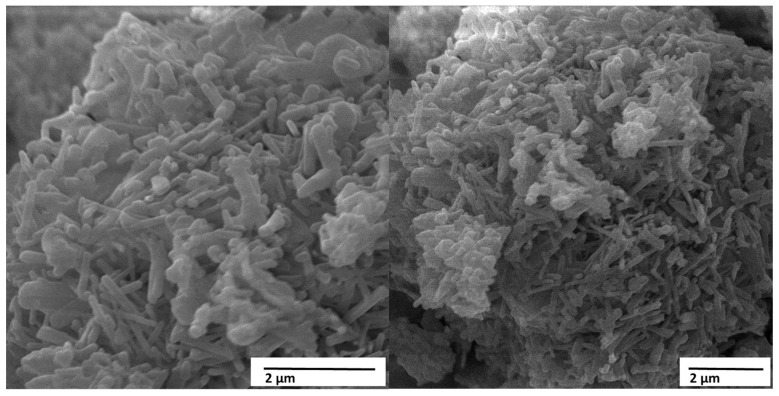
SEM micrographs of the HACRAB sample showing acicular nanocrystals and interconnected porosity. Scale bars = 2 µm (**left**) and 2 µm (**right**).

Figure 10 shows the micrographs obtained by scanning electron microscopy (SEM) of the material HACRAB (HA obtained from crab shell), with magnifications of 20,000× and 30,000×, clearly revealing the crystalline morphology and fine structure of the material.

The images show a set of dense clusters formed by elongated and intertwined crystallites, which organize into compact but still highly porous three-dimensional structures. The observed morphology is typically acicular (needle-shaped) and lamellar, characteristics associated with HAs of biogenic origin and hydrothermal syntheses with high nucleation rates.

At 20,000× magnification, the clusters appear as granular masses composed of fine, elongated crystals, ranging in size from 100 to 300 nm wide and up to 1 µm long, arranged in a random and intertwined manner. This configuration indicates that crystallization occurred under conditions of moderate supersaturation, resulting in preferential growth along the c axis, typical of the hexagonal structure of HA (Ca_10_(PO_4_)_6_(OH)_2_ phase). In the 30,000× image, the crystal packing can be more clearly identified, forming compact clusters permeated by micro- and nanopores, giving the material a hierarchically rough texture, ideal for cell adhesion and ionic interaction in biological media.

The acicular morphology observed in the micrographs is indicative of good crystallinity and structural organization, although the agglomerates indicate that cohesion between fine particles was partially preserved during drying. This morphology contrasts with the HACRAB in Figure 9, which presented spherical and amorphous agglomerates, revealing that, in the case of HACRAB, the HA deposition and formation process was more controlled and oriented, resulting in more defined and uniform crystallites. This behavior is associated with the presence of Ca^2+^ and PO_4_^3−^ ions in proportions close to the HA stoichiometry, and possibly with the hydrothermal conditions used in the synthesis reaction, which favor anisotropic crystal growth.

The presence of intercrystalline pores visible in the micrographs is a positive aspect from a biomedical perspective. This multi-scale porosity (micro and nano) contributes to increased surface area, favoring the adsorption of plasma proteins, the adhesion and proliferation of osteoblasts, and allowing the diffusion of body fluids during integration with bone tissue. Thus, the observed morphology is consistent with the literature, which indicates that HAs with acicular and porous crystals have better bioactivity and osteoconductive capacity compared to granular or dense morphologies.

From a chemical and structural perspective, the presence of well-defined crystals and the apparent absence of amorphous regions suggest that the material underwent an adequate stage of crystalline maturation. This characteristic indicates a thermodynamically stable structure, with a low content of secondary phases such as CaO or Ca(OH)_2_, which could otherwise have been observed as amorphous regions or smooth surfaces. The good definition of the crystals reinforces the idea that the conversion of CaO (obtained from the calcination of crab shells) into HA was efficient, and that the synthesis conditions—temperature, time, and pH—favored the nucleation and oriented growth of the apatite phase.

However, the agglomerated and densely packed appearance of the crystals may limit the dispersibility of the suspended material if used as a precursor for liquid coatings. The formation of these agglomerates suggests strong interaction between the fine particles, possibly through hydrogen bonding or van der Waals forces, which hinders uniform redispersion without the use of ultrasound or suitable dispersants. Therefore, dispersion control becomes critical to ensuring the uniformity of ceramic coatings produced from this powder.

The acicular nanocrystals and interconnected porosity seen in HACRAB are consistent with efficient nucleation on activated Ti–Al–V surfaces. The roughened and oxidized substrate likely provided abundant hydroxyl sites for ionic exchange, promoting oriented crystal growth along the c-axis of HA. This hierarchical microstructure, nano-HA needles on a microrough metallic base, is desirable for biomedical coatings because it maximizes the real contact area and facilitates bone-like apatite precipitation during in vivo integration.

**Figure 11:**
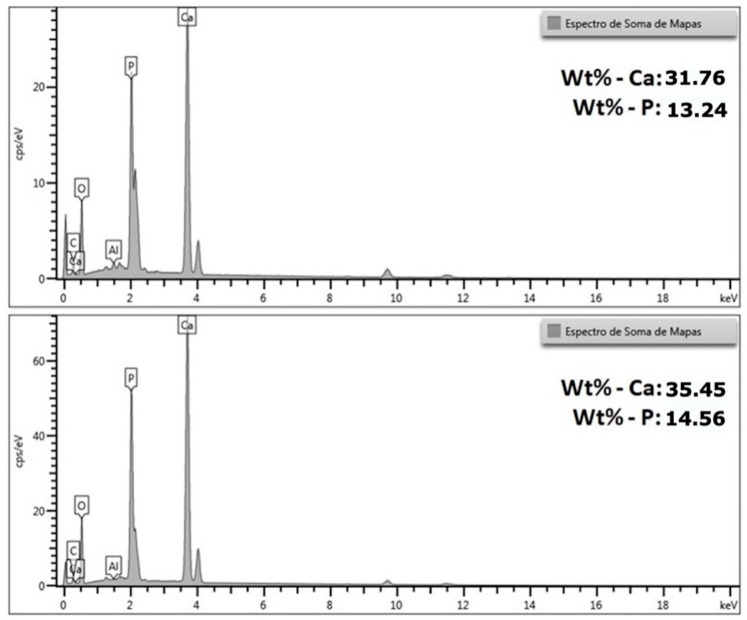
Energy dispersive X-ray spectra (EDS) obtained from the sum of the scan maps of the HACRAB sample.

Figure 11 shows the EDS (Energy Dispersive Spectroscopy) spectra obtained from the map sum analysis of the HA coating produced from the crab exoskeleton.

In both analyses, the characteristic peaks of calcium (Ca), phosphorus (P) and oxygen (O) are observed—main elements of the crystalline structure of HA—in addition to smaller signals of carbon (C) and aluminum (Al), which may be related to the presence of traces of the carbon conductive tape, the analysis environment or the underlying metallic substrate.

The semi-quantitative results obtained indicated contents of 31.76% by weight of Ca and 13.24% of P in the first analyzed region, and 35.45% of Ca and 14.56% of P in the second. From these values, a Ca/P atomic ratio of approximately 1.85 to 1.88 is calculated, slightly higher than the stoichiometric value of pure HA (Ca/P = 1.67). This difference suggests that the synthesized material is rich in calcium and may contain small amounts of secondary phases, such as calcium oxide (CaO) or calcium hydroxide (Ca(OH)_2_), resulting from the incomplete conversion of calcium oxide obtained from the calcination of the crab exoskeleton during the reaction with phosphate.

Despite this slight difference in the Ca/P ratio, the values obtained remain within the expected range for biogenic or partially substituted apatite’s, which typically have ratios between 1.5 and 1.9. This result reinforces that the material formed is predominantly apatitic, which is in full agreement with the SEM micrographs (Figure 9 and Figure 10)—which show an acicular and porous morphology, typical of HA—and also with the XRF analysis (Table 5), which had already indicated an excess of calcium relative to phosphorus. The difference between the XRF and EDS results is expected, since XRF analyzes the total volume of the powder (global compositional average), while EDS performs a localized surface reading, being sensitive to sample heterogeneities and topographies.

From an experimental perspective, some instrumental factors may explain small variations in Ca and P values. The electron beam accelerated at 20 kV penetrates approximately 1–2 µm into the sample, which can generate a mixture of surface and subsurface information, especially in porous materials. Furthermore, irregular topographies—as observed in the micrographs—can cause shadowing and angular detection differences, resulting in small phosphorus undercounts. Another relevant aspect is that, because the EDS was performed without the use of specific calibration standards, the standardless method tends to overestimate calcium and underestimate phosphorus, artificially elevating the Ca/P ratio.

Even with these limitations, the uniformity of the results between the two regions analyzed demonstrates that the coating composition is homogeneous, with small fluctuations expected for biogenic materials. The slight predominance of calcium can be interpreted as an advantage in terms of bioactivity, since Ca-rich materials exhibit greater initial solubility in physiological media and release of Ca^2+^ ions, favoring the nucleation of secondary apatite and the formation of the bonding layer with bone tissue. However, a very high excess of CaO or Ca(OH)_2_ could compromise the chemical and mechanical stability of the coating, causing cracks due to hydration or an increase in local pH in a biological environment.

#### 3.2.4. X-Ray Diffractometry (XRD)

**Figure 12:**
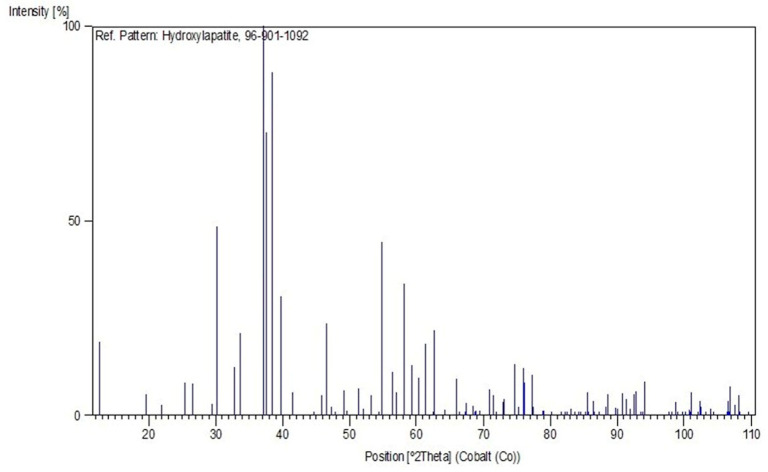
Reference XRD pattern of HA (ICDD 96-901-1092). The diffractogram is plotted as normalized Intensity [%] (y-axis) as a function of 2θ (x-axis), using cobalt radiation. The blue line represents the reference HA pattern used for qualitative comparison with the synthesized material.

Figure 12 shows the X-ray diffractogram (XRD) of the ceramic layer deposited on the metallic substrate, identifying the characteristic peaks of HA according to the ICDD 96-901-1092 reference standard [31].

The XRD pattern of the synthesized HACRAB powder exhibits the characteristic reflections of hexagonal hydroxyapatite (HA), with the main diffraction peaks positioned near 2θ ≈ 25.9°, 31.8°, 32.2°, and 32.9°. These peaks correspond to the (002), (211), (112), and (300) crystallographic planes, respectively, and match well with the reference pattern of stoichiometric HA (ICDD 09-0432). The absence of prominent secondary phases such as tricalcium phosphate (TCP), calcium oxide (CaO), or calcium hydroxide (Ca(OH)_2_) suggests that the wet-chemical process successfully produced a predominantly apatitic structure.

Although the diffraction pattern is consistent with HA, the peaks show slight broadening and minor variations in relative intensity. Peak broadening is typically indicative of nanocrystallinity, coherent domain reduction, and lattice imperfections—features consistent with the SEM observations that revealed submicrometric crystallites. Such broadening is common in biogenic or low-temperature synthesized apatite’s and is not associated with amorphous residues, since no diffuse halo was detected.

The subtle variations in peak intensity and width observed in the diffractogram are consistent with the presence of substituted or biogenic apatite’s, which commonly exhibit lattice distortions associated with carbonate incorporation or trace ionic substitutions. Such modifications are particularly typical of calcium phosphates derived from marine precursors. These substituted structures are well known to enhance biological affinity, increase dissolution rates, and accelerate mineralization kinetics compared to stoichiometric synthetic HA [15,16]. Moreover, natural minor ion substitutions such as Mg^2+^, Sr^2+^, and CO_3_^2−^—frequently present in biogenic apatite’s—are widely reported to promote faster interfacial bonding and improved osteoconductivity in vitro and in vivo [10].

Overall, the crystallographic profile confirms that the HACRAB powder maintains a predominantly HA structure with nanocrystalline features and minor lattice substitutions typical of marine-derived bioapatite’s. These structural characteristics align with the bioactive behavior expected for such materials and support their suitability for coating titanium substrates in biomedical applications.

### 3.3. Overall Discussion

The combined analyses demonstrate that substrate composition, surface treatment, and HA synthesis route act synergistically. It is important to clarify that the synthesis process employed was not a conventional hydrothermal reaction conducted under pressure, but a low-temperature wet-chemical route. The resulting HA layer therefore formed primarily through precipitation and drying, which produces a thin continuous coating with peripheral thickening rather than a dense film grown under autoclave conditions. The α-dominated Ti–Al–V substrate ensured mechanical stability; the thermochemical treatment standardized roughness and increased surface energy; and the aqueous HA deposition produced a continuous, crystalline, Ca-rich layer. Together, these factors resulted in strong qualitative adhesion and a nanostructured, bioactive morphology. Although adhesion was not yet quantified, the microstructural and compositional evidence suggests a chemically bonded interface suitable for further mechanical and biological testing.

## 4. Conclusions

This study demonstrated that biogenic hydroxyapatite synthesized from *Ucides cordatus* crab-shell waste can be successfully deposited onto Ti–Al–V substrates, producing continuous and well-adhered coatings. XRD and FTIR analyses confirmed the formation of a predominantly hexagonal HA phase, while SEM/EDS revealed a nanocrystalline, porous morphology consistent with biogenic apatite’s and a Ca-rich composition typical of marine-derived precursors. The thermochemical surface treatment applied to the titanium substrate proved effective in homogenizing surface roughness within a range favorable for HA nucleation. Cross-sectional microscopy demonstrated coating thicknesses of ~95–98 µm with no evidence of spontaneous delamination during sample preparation, indicating satisfactory qualitative adhesion. Overall, the results validate the technical feasibility of converting crab-shell waste into a functional HA coating material and highlight its potential for sustainable, low-cost biomedical surface engineering. Future work will focus on quantitative adhesion testing according to ISO 13779-2 and on optimizing synthesis routes to refine phase purity and compositional control.

## Figures and Tables

**Table 1:** Chemical composition of the analyzed metal samples in comparison with the reference alloy Ti-6Al-4V according to ASTM F136-18 [28].

**Samples Studied**		
**%Ti**	**%Al**	**%V**
88.75	7.46	3.41
**Ti6Al4V (ASTM F 136-18)**		
**%Ti**	**%Al**	**%V**
Bal.	5.5–6.5	3.5–4.5

Note: “Bal.” indicates “balance,” corresponding to the remainder of the alloy composition.

**Table 2:** Rockwell C hardness (HRC) results of samples A1 and A2 of Ti-6Al-4V alloy, with respective deviations and standard errors, compared to reference values reported in the literature.

	A1	A2	ASM
**Average**	35.07 HRC	35.83 HRC	35–36 HRC
**Std. Dev. (S)**	0.499 HRC	0.923 HRC	-
**Std. Error (e)**	0.157 HRC	0.292 HRC	-

**Table 3:** Results of the average roughness (Ra) measurements of samples A1–A10, obtained for the untreated (ST) and treated (CT) surface conditions. The mean values, standard deviations (S) and standard errors (e) corresponding to each condition analyzed are presented.

	**A1—ST**	**A2—ST**	**A3—ST**	**A4—ST**	**A5—ST**
**Average—Ra (µm)**	0.598	0.954	0.584	0.416	0.702
**Std. Dev. (S)**	0.008	0.047	0.013	0.059	0.042
**Std. Error (e)**	0.005	0.027	0.008	0.034	0.024
	**A6—CT**	**A7—CT**	**A8—CT**	**A9—CT**	**A10—CT**
**Average—Ra (µm)**	0.588	0.586	0.587	0.589	0.587
**Std. Dev. (S)**	0.010786	0.041187	0.017214	0.012055	0.001528
**Std. Error (e)**	0.0062	0.0238	0.0099	0.0070	0.0009

**Table 4:** Average thicknesses of the layers deposited in samples 01 to 04, comparing the conditions without treatment (CP–ST) and with treatment (CP–CT).

	CP–ST (μm)	CP–CT (μm)
Sample 01	90	57
Sample 02	80	131
Sample 03	80	71
Sample 04	129	135
**Average**	95	98

**Table 5:** Chemical composition of the HA layer obtained from the HACRAB material, expressed as a percentage by mass of the main constituent oxides (SrO, P_2_O_5_, K_2_O and CaO).

Composition of the HACRAB			
%SrO	%P_2_O_5_	%K_2_O	%CaO
0.068	33.20	0.063	66.64

## Data Availability

The original contributions presented in the study are included in the article; further inquiries can be directed to the corresponding author.
